# Retrograde access revascularization in chronic limb threatening ischaemia

**DOI:** 10.1186/s42155-026-00786-5

**Published:** 2026-10-14

**Authors:** Yujia Gao, Lorenzo Patrone, Neil Davies, Anthie Papadopoulou, Nick Woodward, Janice Tsui, Mark Portou, Shanka Benaragama, Conrad von Stempel

**Affiliations:** 1 Division of Surgery and Interventional Science, UCL Medical School, University College London, London, UK; 2 Department of Surgery, Vascular and Endovascular Surgery Unit, USL Toscana Centro, San Giovanni Di Dio Hospital, Florence, Italy; 3 Department of Radiology, Royal Free Hospital, London, UK; 4 Department of Vascular Surgery, Royal Free Hospital, London, UK

**Keywords:** Chronic limb threatening ischaemia, Angioplasty, Retrograde access

## Abstract

**Purpose:**

Chronic limb-threatening ischaemia (CLTI) is associated with high rates of major amputation, mortality and comorbidity that can make conventional femoral access challenging or contraindicated; in particular in patients with recent groin surgery, infection, previous aorto-bifemoral stent reconstruction, hostile groins, or unfavourable body habitus. Tibio-pedal arterial minimally invasive retrograde revascularisation (TAMI) provides an alternative access strategy.

**Materials and methods:**

This retrospective single-centre cohort study included 84 TAMI procedures in 81 limbs from 77 patients with CLTI (median age 72 years; 64% male; Rutherford IV 22%; Rutherford V–VI 78%). TAMI was performed because of recent groin surgery/infection in 35% of procedures and patient-related factors in 65%. The primary clinical endpoint was freedom from major adverse limb events (MALE), defined as major amputation or clinically driven target-vessel reintervention (CDTVR). Secondary endpoints included technical success, defined as restoration of continuous inline flow through at least one infrapopliteal artery to the foot with < 50% residual stenosis on completion angiography, and access vessel safety.

**Results:**

Retrograde access was obtained predominantly via the dorsalis pedis (71%) and distal posterior tibial arteries (16%). Technical success was achieved in 66/84 procedures (78%); in 10/84 (12%), TAMI access and lesion crossing were successful but the predefined angiographic endpoint was not met, while eight procedures (10%) were abandoned. Asymptomatic access vessel spasm was documented in 35/84 procedures (42%) that had resolved by 24 h. Among 68 patients (71 limbs) with longitudinal follow-up, median follow-up was 150 days (IQR 76–322). CDTVR occurred in 22/71 limbs (30%) and major amputation in 14/71 (20%); 25/68 patients (37%) died. Kaplan–Meier estimated freedom from MALE was 57.9% (95% CI 43.9–69.6%) at 6 months and 51.2% (95% CI 36.0–64.5%) at 12 months. In multivariable Cox regression analysis, none of the evaluated covariates was significantly associated with MALE.

**Conclusion:**

In this selected, anatomically complex CLTI cohort, TAMI was a feasible alternative access strategy when conventional femoral access was challenging or not feasible, with a low observed rate of clinically significant pedal access complications.

## Introduction

Chronic limb-threatening ischaemia (CLTI) represents the end-stage of peripheral arterial disease (PAD) and requires coordinated multidisciplinary management to prevent major amputation and reduce mortality [1]. Conventional endovascular revascularisation is typically performed via antegrade ipsilateral common femoral artery (CFA) access or a contralateral retrograde crossover technique. However, these approaches may be impractical or contraindicated in patients with recent groin surgery, prior aortic bifurcation reconstruction, severe obesity [2], or limb contractures.

Tibio-pedal arterial minimally invasive (TAMI) access is a primary retrograde pedal arterial approach in which diagnostic angiography and endovascular intervention can be performed from distal tibio-pedal access. In selected complex cases, an additional femoral access may be used to facilitate crossing or re-entry. With the advent of thin-walled sheaths and low-profile devices, a broad range of endovascular techniques can be delivered through this approach, with potential advantages in maintaining luminal wire position when crossing chronic total occlusions.

This study aimed to assess the technical feasibility, access vessel safety and clinical outcomes of TAMI as an alternative access strategy in patients with CLTI in whom conventional femoral access was challenging or not feasible at a tertiary vascular centre.

## Methods

### Study design

This was a retrospective, single-centre cohort study of patients undergoing TAMI endovascular revascularisation for CLTI between January 2019 and January 2025. Clinical and longitudinal follow-up data were extracted from electronic medical records. Procedural and imaging data were reviewed using PACS and operative reports.

### Ethics approval and consent to participate

Formal Research Ethics Committee approval and the requirement for informed patient consent in the study were waived in accordance with institutional policies governing retrospective analyses of anonymised clinical data.

### Consent for publication

Written consent for publication of images was obtained.

The datasets analysed during the current study are available from the corresponding author on reasonable request.

Anatomical complexity was classified using the Global Vascular Guidelines on the Management of Chronic Limb-Threatening Ischaemia (GLASS) classification [1]. All cases and imaging were reviewed by a single consultant vascular interventional radiologist.

The primary clinical endpoint was freedom from major adverse limb events (MALE), with MALE defined as major (above-ankle) amputation or clinically driven target-vessel reintervention (CDTVR). CDTVR was defined as repeat endovascular or surgical revascularisation of the treated target vessel or segment prompted by recurrent or persistent CLTI symptoms or failure of wound healing. Secondary endpoints included technical success, defined as restoration of continuous inline angiographic flow through at least one infrapopliteal artery to the foot and with < 50% residual stenosis on completion angiography.

Patency surveillance was performed using duplex ultrasound at 2–6 weeks post-procedure, with subsequent clinical and podiatric follow-up guided by wound healing and symptoms.

### Patient selection

TAMI was selected when anatomical or patient-related factors precluded standard femoral access and pre-procedural imaging demonstrated predominantly superficial femoral and/or popliteal artery disease. Absolute contraindications to antegrade femoral access included recent groin cut-down (< 6 weeks), extensive CFA scarring, or prior aortic bifurcation stent-graft reconstruction. Relative contraindications included severe anterior-wall CFA calcification, recent failed femoral access, groin infection, morbid obesity with a pannus and fixed hip contractures.

### TAMI procedural technique

Written procedural informed consent was obtained in all cases. Pre-operative and peri-procedural duplex ultrasound was used to identify a suitable pedal artery (> 1.0 mm diameter with a detectable spectral waveform). The dorsalis pedis (at the level of the tarsal neck), posterior tibial artery (at the level of the medial malleolus) and peroneal artery (proximal to its bifurcation overlying the distal fibular metaphysis) were interrogated using B-mode ultrasound to characterise calcification burden and vessel calibre; colour and pulsed-wave Doppler were used to confirm patency. A single patent tibial run-off vessel was considered a relative contraindication to TAMI. In 10 procedures involving a single run-off vessel, an occluded pedal vessel was accessed in four to avoid instrumenting the only patent run-off vessel.

Ultrasound-guided transaxial puncture was performed using a 22-gauge micro-access needle (Terumo, Tokyo, Japan) followed by insertion of a nitinol 0.021inch guidewire under direct ultrasound visualisation and subsequently a thin-walled 5–7 Fr sheath (outer lumen equivalent 4–6 Fr, Slender sheath, Terumo, Tokyo, Japan).

Systemic anticoagulation was achieved with intravenous heparin targeting an activated clotting time > 200 s. Retrograde ‘reflux’ angiography was performed via hand-injected contrast reflux. Lesion crossing was performed via dedicated 0.018 inch, 300 cm guidewires (Command-18 ST, Abbott (Abbott Park, IL, USA), Glidewire Advantage, Terumo (Tokyo, Japan), Astato 30, Asahi (Aichi, Japan)) and crossing catheters (CXI 2.6fr 135 cm and 4fr 90 cm Cook (Bloomington, Indiana, USA)). Vessel preparation included intravascular lithotripsy for PACSS ≥ 3 lesions (Shockwave, Santa Clara, CA, USA) in three cases, followed by angioplasty with plain (Sterling, Boston Scientific Corporation (Marlborough, MA, USA)) or drug-coated balloons (IN.PACT (Medtronic, Dublin, Ireland)) for femoral-popliteal lesions. Bail-out femoral-popliteal stenting was performed in three cases with (Supera, Abbott (Abbott Park, IL, USA)).

**Fig. 1:**
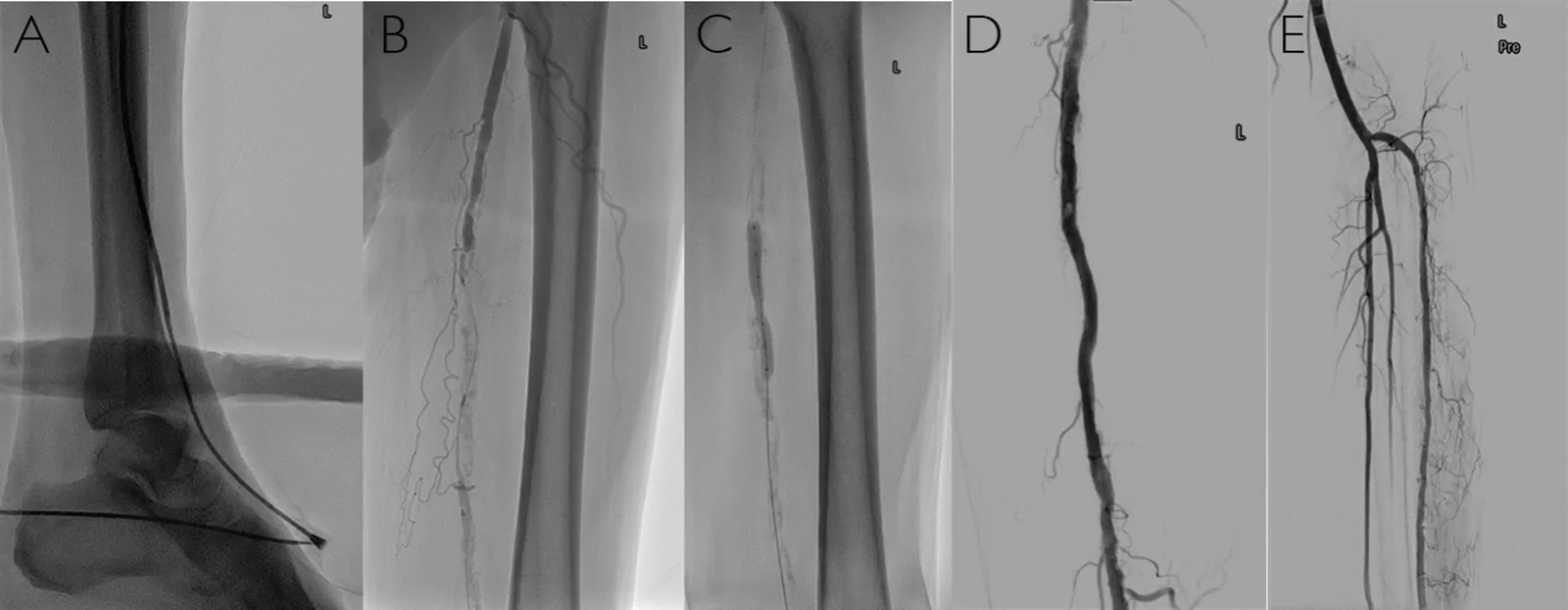
Selective images from a TAMI angioplasty of a GLASS stage 1 lesion characterised by a 12 cm SFA CTO. **A** DPA access and 5Fr sheath in situ with fluoroscopic selected image following retrograde contrast reflux showing a patent ATA. **B** Angiographic still non-subtracted to demonstrate the dense calcification in the chronic total occlusion in the mid SFA. Retrogradely placed crossing catheter tip seen in the proximal SFA. **C** Fluoroscopic still image showing a 7 mm × 6 cm intravascular lithotripsy balloon inflated in the chronic total occlusion. **D**, **E** Digital subtraction angiograms showing restored flow in the SFA (woven nitinol stent 6.5mm×10cm has been deployed across the occluded segment) and into three infrageniculate vessels

Haemostasis at the TAMI access site was achieved with manual compression for five minutes. See Fig. 1.

### Statistical analysis

Statistical analysis was performed with SPSS (IBM SPSS Statistics, Version 31.0, IBM Corp). The primary clinical endpoint was MALE (major amputation or CDTVR). Limb-related outcomes were also reported separately. Time-to-event was defined as the interval from the index TAMI procedure to the first composite event, with event-free patients censored at their last clinical follow-up. Multivariable Cox proportional hazards regression was performed using the Enter method. Covariates were selected a priori based on clinical relevance and included age, diabetes mellitus, CKD stage ≥ 3, smoking history and anatomical complexity (GLASS stage III versus I–II). The proportional hazards assumption was assessed using Schoenfeld residuals, both for individual covariates and globally. Adjusted hazard ratios (HRs) with 95% confidence intervals (CIs) were reported. In patients undergoing a repeat procedure with TAMI on the index limb, the repeat procedure was considered a CDTVR event.

## Results

### Patient characteristics

**Fig. 2:**
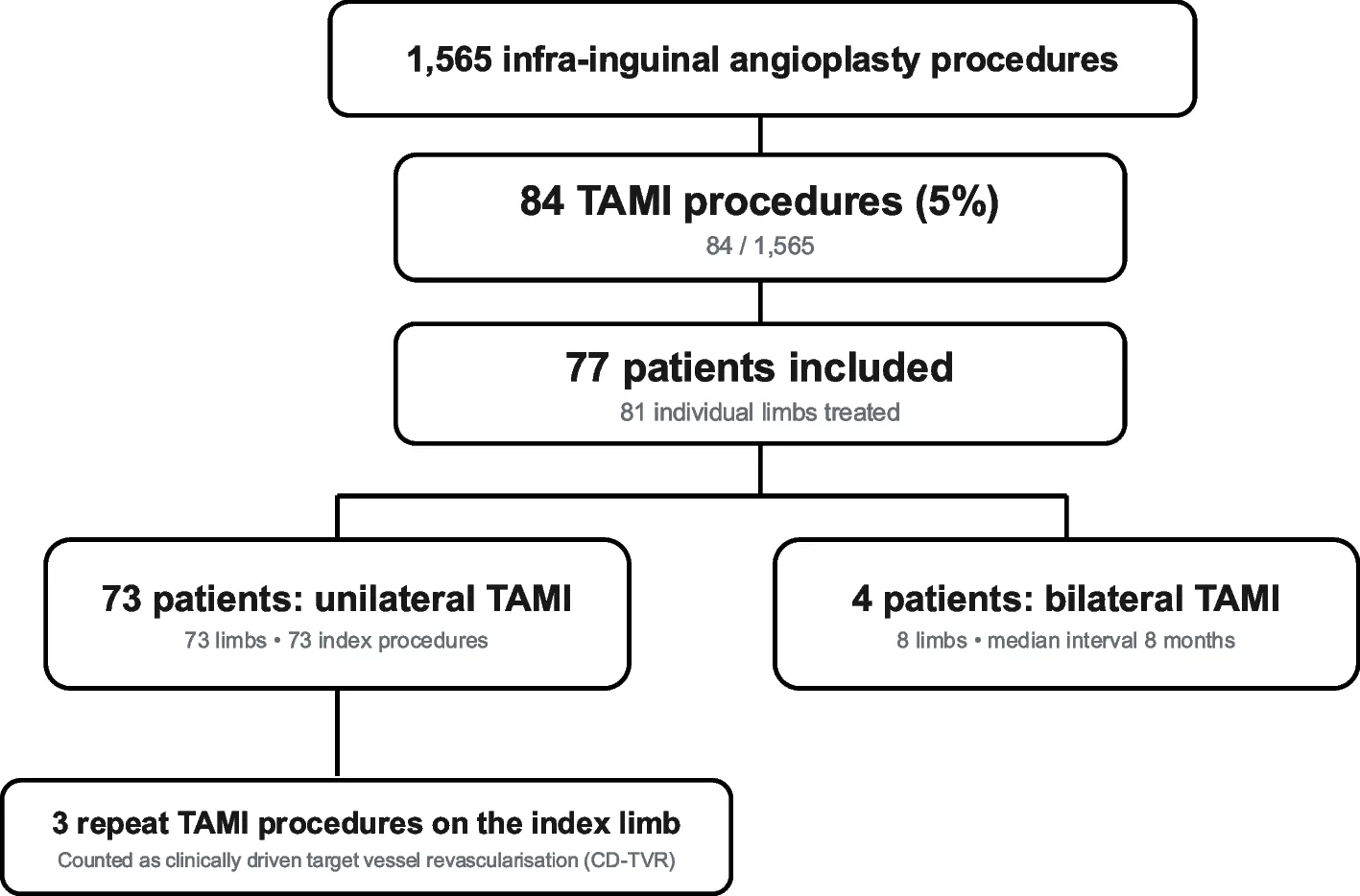
STARD diagram showing included patients, limbs and procedures

TAMI accounted for 5% (84/1565) of all infra-inguinal angioplasty procedures performed during the 5-year study period (January 2019–January 2025). The study included 77 patients in whom 81 individual legs underwent TAMI angioplasty. Of these, 73 patients underwent unilateral TAMI, while four patients underwent TAMI in both limbs, with a median interval of 8 months between sides. Three patients subsequently underwent repeat TAMI of the index limb; these repeat interventions were considered to meet the definition of clinically driven target vessel revascularisation (CDTVR). Thus, 84 TAMI procedures were performed across 81 limbs in 77 patients (see Fig. 2).

The median age of patients was 72 years with 64% male.

The multilevel complexity of disease with a high burden of chronic total occlusion and calcification was reflected in the GLASS scores of the 81 limbs included: stage I in 9 legs (11%), stage II in 17 (21%) and stage III in 55 (68%) (see Table 1).

**Table 1:** Patient comorbidities and disease staging

Patient details *n =* *77*			
Comorbidities	DM	48	62%
	CKD 3 +	15	19%
	Smoking history	38	49%
GLASS Stage		Number of Limbs	Percentage
	Stage I	9	11%
	Stage II	17	21%
	Stage III	55	68%

*Abbreviations*: *DM* Diabetes mellitus, *CKD* Chronic kidney disease, *GLASS* Global Vascular Guidelines on the Management of Chronic Limb-Threatening Ischemia [1]

Rutherford category 4 was present in 17/77 patients (22%), category 5 in 52/77 (68%) and category 6 in 8/77 (10%).

### Procedural details

Forty per cent of cases involved limbs that had undergone endovascular intervention within the preceding 12 months for the same lesion (antegrade or up-and-over approaches).

Median procedure duration was 125 min (IQR 120–150), and median dose-area product was 6.07 Gy.cm^2^ (IQR 2.78–15.35).

Seventy-two procedures (86%) were performed under local anaesthetic with the addition of intravenous conscious sedation (53 cases; 63%) and combined with regional anaesthesia with popliteal nerve blocks (18 cases; 21%). General anaesthesia was required in 12 cases (15%).

Across 84 procedures, TAMI was indicated because of recent (< 6 weeks) groin cut-down in 26 (31%), groin infection in 3 (4%), fixed contracture preventing standard positioning in 18 (21%), morbid obesity in 34 (40%) and not documented in 3 cases (4%).

### Technical outcomes

Of 84 procedures, technical success with restoration of continuous inline flow through at least one infrapopliteal artery to the foot with < 50% residual stenosis was achieved in 66 (78%). Of those, three (4%) required an additional antegrade ipsilateral SFA puncture with placement of a microsheath (4fr S-MAK, Merit medical, UT, USA) and an 0.018inch wire to assist luminal re-entry during long CTO recanalisation; these were retained in the cohort as bidirectional TAMI-assisted procedures, with definitive vessel-preparation and angioplasty/stenting performed from TAMI access; these cases are reported as a subgroup in Table 2. In 3 cases bail-out bare-metal stents were deployed in the distal CFA-proximal SFA (7.5 mm x 80 mm Supera) and in the mid SFA/P1 segment of the popliteal (5.5 × 100 mm Supera) due to significant flow-limiting dissection despite prolonged angioplasty attempts.

**Table 2:** Procedural details

Procedural details *n =* *84*				
Sheath	Sheathless		1	1%
	4-Fr		4	5%
	5-Fr		76	90%
	6-Fr		2	3%
	7-Fr		1	1%
Access vessels	Single	DPA	60	71%
		PTA	13	16%
		Distal peroneal artery	1	1%
	Dual	DPA + PTA	7	8%
		DPA + antegrade SFA wire	3	4%
Treatment target	SFA		59	70%
	Popliteal		42	50%
	ATA		29	35%
	PTA		10	12%
	CFA		9	11%

Denominator is procedures (*n =* 84). Treatment targets are not mutually exclusive. DPA + antegrade SFA cases represent bidirectional TAMI-assisted procedures

*Abbreviations*: *ATA* Anterior tibial artery, *PTA* Posterior tibial artery, *SFA* Superficial femoral artery, *CFA* Common femoral artery

In 10 procedures (12%) angiographic technical success was not met, either due to residual stenosis > 50% and/or poor infrageniculate run-off, but successful lesional crossing was performed. Eight procedures (10%) were abandoned because of failure to cross (*n =* 6), pain intolerance (*n =* 1), or delirium (*n =* 1).

### Complications

On table duplex was performed in all cases and this demonstrated asymptomatic access vessel spasm in 35/84 procedures (42%) with resolution and patent access vessel on formal duplex follow-up (including the 3 patients with 6/7fr sheaths) by 24 h. Overall, delayed formal imaging follow-up with either Duplex or CT angiogram was available in a total of 50 (62%) of limbs at a median of 184 days (IQR 54–668); in four cases where an occluded vessel had been accessed, this remained occluded on follow up and in 46 cases the access vessel remained patent. Formal imaging follow-up of TAMI access vessels in 25 limbs (31%) was not available, with no reported clinical manifestation of occlusion; a further 6 limbs (7%) underwent major amputation for progressive CLTI before subsequent access vessel imaging and the cause for amputation was not documented as a complication of TAMI access.

One patient developed a groin haematoma from a leak at a recent CFA endarterectomy patch; this was classified as a procedural complication unrelated to the pedal access site and resolved with a pressure dressing.

### Clinical outcomes

Within 30 days of the index procedure, 1/77 patients died from a cause not considered procedure-related (myocardial infarction); 6/81 limbs (7%) experienced MALE: one major amputation (1%) and 5 CDTVR events (5/81, 6%).

Longitudinal follow-up beyond 30 days was available for 71 limbs (68 patients) (88%), who formed the survival-analysis cohort. Median follow-up was 150 days (IQR 76–322). Among these 68 patients, 25 died (37%) at a median of 268 days (IQR 100–549), predominantly from ischaemic heart disease and complications of diabetes.

Among the 71 limbs included in longitudinal follow-up, 31 (44%) experienced at least one MALE event. Fourteen (20%) underwent major amputation and 21 (30%) underwent CDTVR; four limbs underwent CDTVR before subsequently undergoing major amputation. In three cases, CDTVR consisted of a repeat TAMI procedure.

### Predictors of outcome

Kaplan–Meier analysis used the primary composite endpoint of major amputation or CDTVR (MALE), with event-free patients censored at last clinical follow-up. Estimated freedom from MALE was 57.9% (95% CI 43.9–69.6%) at 6 months and 51.2% (95% CI 36.0–64.5%) at 12 months, with 24 and 13 patients at risk, respectively. Estimated freedom from major amputation was 75.2% (95% CI 60.8–84.9%) at both 6 and 12 months, with 28 and 15 patients at risk, respectively. There was no significant difference in MALE-free survival between patients with GLASS stage III disease and those with GLASS stage I–II disease (log-rank χ^2^ = 0.046, *p =* 0.830). Survival analysis was performed including 31 MALE events among the 71 limbs of 68 patients included in the survival analysis. On multivariable Cox proportional hazards regression, none of the evaluated covariates were significantly associated with MALE: CKD stage ≥ 3 had the largest point estimate for increased hazard (HR 1.81, 95% CI 0.81–4.03; *p =* 0.148); GLASS stage 3 was associated with an HR of 1.25 (95% CI 0.56–2.79; *p =* 0.590), age (HR 1.01 per year, 95% CI 0.97–1.04; *p =* 0.706), diabetes mellitus (HR 1.03, 95% CI 0.47–2.27; *p =* 0.944) and smoking (HR 0.94, 95% CI 0.44–2.03; *p =* 0.878) were not significantly associated with MALE (see Table 3). The overall multivariable model was not statistically significant (χ^2^ = 2.83, 5 df; *p =* 0.726).

**Table 3:** Cox regression variables

*Variable*	*Adjusted HR*	*95% CI*	*p*
Age	1.01	0.973–1.041	0.706
Diabetes	1.03	0.466–2.273	0.944
CKD ≥ 3	1.81	0.810–4.026	0.148
Smoking	0.94	0.437–2.030	0.878
GLASS 3	1.25	0.558–2.790	0.590

The proportional hazards assumption was assessed using Schoenfeld residuals. No evidence of violation for any individual covariate (all *p >* 0.05) or for the model globally (*p =* 0.825) was identified.

## Discussion

In this study of patients with CLTI and anatomically complex disease (68% GLASS stage III), TAMI provided an alternative access strategy for endovascular revascularisation when conventional femoral access was not feasible. Technical success, as defined as restoration of at least one infrageniculate run-off vessel without significant residual stenosis, was achieved in 78% of procedures. In this study access vessel spasm was encountered in 42% of cases but in all cases a follow-up duplex at 24 h showed resolution; in total 62% of the cohort had formal delayed imaging follow-up at a median of 184 days, which confirmed access vessel patency in all cases where a patent pedal vessel was accessed (46/50 92%) and in 4 cases where an occluded pedal vessel was accessed at the index procedure remained occluded on follow-up imaging.

Previous TAMI series in mixed PAD populations have reported technical success rates exceeding 90% and access vessel occlusion rates of approximately 3–7.5% [3–8]. The lower technical success observed in the present cohort (78%) may reflect its restriction to CLTI and the high burden of chronic occlusive and heavily calcified disease. Procedure duration was also longer than in previously published TAMI series (median 125 min versus reported averages of approximately 64–76.7 min), again potentially reflecting case complexity. Formal imaging confirming access vessel patency was not measured in all cases. During longitudinal follow-up of a survival analysis cohort of 71 limbs, 14 limbs (20%) underwent major amputation and 21 limbs (30%) underwent CDTVR.

The TAMI approach offers practical advantages over traditional access in selected cases, particularly in those with multiple recent ipsilateral interventions and comorbidities such as obesity, sleep apnoea and hip contracture. TAMI offers practical advantages of retrograde access, including improved luminal crossing of CTO, improved device pushability [8] and the creation of a retrograde flap during subintimal crossing which is oriented opposite to blood flow, reducing the risk of significant flow-limiting dissections. Furthermore, haemostasis is rapid with the TAMI approach and can be easily achieved with 5 min of manual compression, potentially followed by the application of a compression bandage even in a restless or noncompliant patient with the added advantage of an easy site to monitor by recovery staff. Despite its advantages, TAMI presents technical limitations: careful planning of the target-path artery is required with work-up duplex and cross-sectional imaging; smaller sheath sizes can restrict the use of some devices and angiography is performed by injecting contrast against the direction of flow with a ‘reflux’ of contrast which can lead to poor distal run-off visualisation. SFA, popliteal and the accessed infrapopliteal vessel lesions are readily treated with TAMI but treatment of additional infrapopliteal vessel generally requires a second puncture. Twelve percent of patients in this study had a single infrageniculate run-off vessel, a setting in which pedal access requires particular caution; in such cases an occluded pedal vessel was preferred as primary TAMI access.

This study is limited by its retrospective, single-centre design and the absence of a contemporaneous femoral access comparison group. TAMI was used in a highly selected population in whom conventional access was challenging or not feasible, introducing substantial selection bias and limiting generalisability. Longitudinal follow-up was available for 68/77 patients (88%) and median follow-up was 150 days, limiting conclusions regarding longer-term limb outcomes and survival. Furthermore formal ultrasound follow-up of the access vessel was only available in 62%. Future prospective multicentre studies with prespecified access-site surveillance and comparative cohorts are required to define patient selection and longer-term outcomes.

## Conclusions

TAMI access for revascularisation in carefully selected patients with CLTI provides a feasible alternative strategy when conventional femoral access is challenging or not technically feasible.

## Abbreviations

PAD: Peripheral arterial disease
DM: Diabetes mellitus
CKD: Chronic kidney disease
GLASS: Global Vascular Guidelines on the Management of Chronic Limb-Threatening Ischemia
CLTI: Chronic limb-threatening ischaemia
TAMI: Tibio-pedal arterial minimally invasive retrograde revascularization
MALE: Major adverse limb events
CDTVR: Clinically driven target vessel reintervention
CFA: Common femoral artery
HR: Hazard ratios
Cis: Confidence intervals
